# "The Hospital" Medical Book Supplement.—No. VII

**Published:** 1908-05-23

**Authors:** 


### "THE HOSPITAL" MEDICAL BOOK SUPPLEMENT.—No. VII.

CONTENTS.

**Medicine—**

Functional Nerve Diseases ... ... ... ... ... ... 207

Medicine in the British Isles ... ... ... ... ... ... 207

Proceedings of the Royal Society of Medicine ... ... ... 208

**Gynæcology and Obstetrics—**

Rotunda Midwifery for Nurses and Midwives ... ... ... 208

A Short Manual for Monthly Nurses ... ... ... ... 208

**Materia Medica and Therapeutics—**

The Book of Receipts ... ... ... ... ... ... ... 208

**First Aid—**

First Aid to the Injured ... ... ... ... ... ... .. 209

"First Aid" to the Injured and Sick: an Advanced Ambulance Handbook ... ... ... ... ... ... ... ... ... 209

**Hygiene—**

Consumption: Home Treatment and Rules for Li[illegible]ing ... ... 209

School Hygiene ... ... ... ... ... ... ... ... 209

Third Annual Report of the Henry Phipps Ins[illegible]te for the Study, Treatment, and Prevention of Tubercul[illegible]is ... ... 209

**Bacteriology—**

A Laboratory Handbook of Bacteriology ... ... ... ... 210

**Stray Notes—**

The Romance of Medicine ... ... ... ... ... ... 210

Alcohol ... ... ... ... ... ... ... ... ... 210

The New Ethics ... ... ... ... ... ... ... ... 210

### MEDICINE.

FUNCTIONAL NERVE DISEASES. By A. T. SCHOFIELD, M.D. With three diagrams. Pp. 324. (London: Methuen and Co. 1908.)

It is difficult to take this book seriously or to guess for what class of reader it is intended. Certainly any medical man who reads it in the hope of enlightenment as to the diagnosis of functional nervous maladies will be quickly undeceived. On the other hand he may derive much innocent amusement from its voluble and high-sounding periods. The introductory chapter—in fact a very large proportion of the book—consists mainly in a collection of ill-digested quotations from other writers. The compiler has apparently wandered from book to book, armed with scissors and paste, and the result is totally devoid of continuity. Between his crops of quotations we find many rotund phrases which could only impress an ill-instructed reader; for example, (p. 20), "electrons seem as clearly the meeting-point of force and matter as neurons are of mind and brain." There is a crude and inaccurate diagram of the nervous system, which is divided into three main divisions—namely, the Cortex or Upper Brain (the seat of the spirit), the Mid-Brain (the seat of the soul), and the Medulla or Hind-Brain (the seat of the body). In this diagram the Mid-Brain includes not only the basal ganglia but also part of the frontal and occipital lobes below an arbitrary line, whilst the Medulla appears to include the cerebellum. The author himself seems to have a lingering suspicion that he has fallen short of perfection, for beneath it we can decipher, in minute type, the legend that "this is purely diagrammatic; there are no such well-defined centres or arcs"! The succeeding chapters profess to give an account of the ætiology and symptomatology of neurasthenia and hysteria, but they are full of the grossest inaccuracies, and altogether beneath criticism.

The one moderately good chapter in the book is that devoted to a description of the rest-cure. The chapter on "Other functional nerve-diseases" is on the same plane as those on hysteria and neurasthenia.

Finally, after a readable chapter on quackery and the value of psycho-therapeutics, we come to a voluminous index. This alone is a fount of innocent joy to the reader. Not only do we find references to "Diagnosis for patient," and "Puberty, danger of," but we are also guided by the alluring title of "Lady and dish" to an interesting tale, which, in case our readers may not secure a copy of the book for themselves, we take the liberty of quoting afresh: "A lady saw a heavy dish fall on her child's hand, cutting off three of the fingers. She felt great pain in her hand, and on examination the corresponding three fingers were swollen and inflamed. In twenty-four hours incisions were made and pus evacuated."

MEDICINE IN THE BRITISH ISLES. By NORMAN MOORE, M.D., F.R.C.P., Physician to St. Barth[illegible]lomew's Hospital. (Oxford: The Clarendon Press, 1908.)

MEDICAL history is, we believe, a subject included in the syllabus for the London M.D., and a modicum of knowledge in it may also be demanded from candidates for other higher examinations. But further we have not progressed, though the recent creation of the Fitzpatrick Lectures of the Royal College of Physicians gives hope for the regeneration of permanent interest in a subject that is as instructive as it is absorbing. Dr. Norman Moore, who is well known as a devout student of this most attractive subject—the history of medicine—publishes in a form which renders them available for reference his Fitzpatrick Lectures on "The History of the Study of Medicine in the British Islands." It is an unpretentious work, but it gives evidence of much research, wide reading, and, what is after all the main count, sympathetic treatment. There are two methods of treating medical history, the sharply critical, such as Professor Haeser loved, and the purely narrative, such as Siebold attempted in his monumental "History of Obstetrics," a work, by the way, that is comparatively little known to English students. Dr. Moore has followed neither system in its entirety, though his lectures approach more the narrative than the critical method, but has attempted to combine the two. The result is what one would have expected from so sympathetic and erudite a student. The brief sketches are exquisite little biographies, and the series is almost a complete one. The only omission (and it is one at which we confess we are somewhat surprised) is a note on Dr. van den Boon, whose influence on the study of medicine in England and Ireland was certainly not less than that of Dr. Mead. For the rest one can only express admiration for the patient investigation, the original research, and the enthusiasm that have given us these lectures.

It would be difficult to single out any part of these lectures for special comment. The whole book ought to be read, and once it has been read we feel sure it will not be relegated to an obscure shelf, but retain an honourable place, handy for future reading. The full *résumé* of Mirfield's work and life, which Dr. Moore has given in the first few lectures, claims, perhaps, a distinctive mention. The works of this old author are rare and practically unknown, and for that reason he has not received the attention he deserved at the hands of medical historians. Mayerne's manuscript notes on the health of James I., which Dr. Moore publishes in the Appendix from the MS. existing in the Sloane Collection at the British Museum, are equally interesting. A third feature is Harvey's MS. notes on Galen, of which Dr. Moore promises a full account in a future number of the St. Bartholomew's Hospital reports.

Proceedings of the Royal Society of Medicine. Volume I. No. 1. (London: Longmans, Green and Company. November 1907. Price 7s. 6d. to non-subscribers.)

The Royal Society of Medicine, as is now widely known, consists of an amalgamation of over a dozen other societies, each of which formerly had its own annual publication of transactions. Instead of the latter appearing once a year, the combined societies now issue their proceedings monthly in the form of a handsomely printed volume of large 8vo. size. Instead, therefore, of having to wait nearly a year before a paper read before a Society appears in print the issue takes place in a month; and not only this, for whereas formerly a separate subscription was required before one was entitled to the transactions of a particular Society, a single subscription now obtains for one the papers that have been read before all the combined societies. The number before us, for example, contains accounts of the patients shown before the Clinical Section of the Royal Society of Medicine, formerly the Clinical Society; notes of the cases shown before the Dermatological Section, formerly the Dermatological Society; papers read before the Electro-Therapeutical, the Epidemiological, the Medical, the Neurological, the Obstetrical, the Gynæcological, the Odontological, the Pathological, the Surgical, the Therapeutical, and the Pharmacological Sections of the Royal Society of Medicine.

The papers are all good, for they have all been before a committee for approval before they could be read. The value of a monthly publication of this kind and style is very obvious. A single publication containing all the best and newest work in every branch of medicine, surgery, gynæcology, and special subjects is now obtainable by every Fellow of the Royal Society of Medicine for the inclusive sum of three guineas per annum. Above and beyond this, London may feel proud to think that it is sending to other countries in this way the collected work of its best intellects. It was almost impossible for every foreign library to take in all the publications of all the societies that used to exist in London; the result was that much of what was done here remained unknown abroad. Now that so many different publications have been rolled into one, this can no longer be the case. It is only to be regretted that still more societies cannot be amalgamated with the Royal Society of Medicine, in order to diminish their separate publications still further.

### GYNÆCOLOGY AND OBSTETRICS.

Rotunda Midwifery for Nurses and Midwives. By G. T. Wrench, M.D., late Assistant Master Rotunda Hospital. (Oxford Medical Publications; Henry Frowde, Hodder and Stoughton. Pp. 324. Price 6s. net.)

In this volume are contained the outlines of midwifery as taught in Dublin, set forth in such a way as to be of use to those preparing for the examinations of the Central Midwives Board, whose rules and regulations, as they at present stand, are given in full in an appendix. For the most part the instruction is simple, clear, and authoritative. It is expressed in short, incisive sentences, admirably free from the technicalities which so often form stumbling-blocks to nurses studying medical subjects. The provinces of the midwife and the doctor are clearly demarcated, and a thoroughly common-sense and practical note is struck throughout. At the same time, teachers and pupils in this country must recognise that here and there the teaching of Dr. Wrench is not that admitted on this side of the Irish Channel. In other words, nurses studying for the C.M.B. certificate cannot rely wholly on this book without the supervision of an experienced teacher to point out these occasional divergencies. As long as the author confines himself to the pithy dogmatic sentences at which he excels there is little fault to find with the manner of his discourse, though the repeated references to the "conduction" of labour are irritating; but when he ventures upon longer sentences, punctuation, diction, even grammar, are somewhat to seek. That would not greatly signify did it not at times actually obscure the meaning. The quality of the illustrations is uneven—some are good, others very poor, and not all are necessary. We mention these points not because they outweigh the very great merits of the work, but because they could so easily be remedied, and because in other respects Dr. Wrench's manual is so eminently suited for its purpose.

A Short Manual for Monthly Nurses. By C. J. Cullingworth, M.D., F.R.C.P. Sixth Edition, revised and enlarged. (London: J. and A. Churchill. Pp. 128. Price 1s. 6d. net.)

With the development of the midwife and of the Central Midwives Board there has necessarily ensued some diminution in the status and importance of the purely "monthly nurse." But the issue of the late Dr. Cullingworth's excellent treatise in its new form is a timely reminder that that useful personage is by no means entirely superseded or dispensable. It is no disparagement of the more highly-trained holder of the Central Midwives Board certificate to say that a nurse who has mastered the letter and spirit of this book will prove as useful an auxiliary in the accouchement chamber as the obstetric physician can possibly desire.

### MATERIA MEDICA AND THERAPEUTICS.

The Book of Receipts. By E. W. Lucas, F.I.C., F.C.S. Pp. 451. Plates x., figs. 20. Demy 8vo. Eleventh edition. (London: Messrs. J. and A. Churchill. Price 7s. 6d. net.)

The title of this book might suggest that it was chiefly concerned with cookery, but this is by no means the case. There is hardly a single class of the community that will not find it useful in many of the little things of every-day life. The veterinary surgeon, farmer, and animal fancier will find all the commoner ailments of horses, cattle, sheep, swine, dogs, cats, poultry, and birds briefly discussed, along with the formulæ for the boluses, lotions, powders, and so forth appropriate to the treatment of each. The housewife will find the chapter upon Domestic Formularies a most helpful addition to Mrs. Beeton, if she wishes to look up the recipes for making artificial fruit essences, sauces of various sorts (from mayonnaise to Worcester), food preservatives, liqueurs, chutneys, pastes, and so forth. A special chapter is devoted to the composition of vermin-killers, fly-papers, and similar things, freezing mixtures, furniture polishes, leather revivers, boot powders and polishes, harness and saddle polishes, metal polishes, varnishes, wood stains, laundry preparations, lacquers, pastes, inks, sealing wax, and insecticides.

Photographers will find twenty pages devoted to the various reagents used in their art. The formulæ and composition of innumerable proprietary articles, toilet soaps, hair oils, corn plasters, pastilles, pomades, and skin lotions are fully given. The medical practitioner will find useful stores of information as to the composition of all manner of medicaments, amongst which we may mention particularly such things as are of service in minor medicine—chilblain applications, powders for ill-smelling perspiring feet, bath powders, tooth powders and pastes, insect-bite applications, smelling salts, snuffs, and a hundred and one similar things. Perfumes and their formularies have a chapter to themselves. There are brief but useful summaries of the methods of analysis of urine, water, and milk; tables of the composition of the best-known spa waters; lists of synonyms; tables of incompatibles; tables of poisons and their antidotes; lists of the principal chemical substances used in pharmaceutics, together with their chemical formulæ and their molecular weights; tables of solubilities and substances that are used in medicine, and abundance of useful information upon all sorts of things which cannot but be of service to a doctor both in his private and in his professional capacities. There is an excellent index. Notwithstanding the great bulk of material in the volume the latter is little more than an inch thick, and yet the printing is of good size and it is easy to find what one is looking for in it. We are not surprised that the book has reached its eleventh edition.

### FIRST AID.

First Aid to the Injured. Six ambulance lectures. Translated from the German of Dr. Friedrich von Esmarch, Professor of Surgery at the University of Kiel, by H.R.H. Princess Christian. Seventh Edition. Pages xv+138, with 46 Illustrations. (London: Messrs, Smith, Elder and Co. Price 2s. net.)

The popularity of this little work is shown by the fact that this is its seventh edition in English. The original book in German attained its 21st edition in 1906. There are several small works of the kind in use in England, but none is simpler, more straightforward, nor more convenient as to size and print, than that before us. The illustrations of the methods of using extemporised bandages, of performing artificial respiration, and so forth, are clear and easy to follow. The kinds of things dealt with are: brief outlines of the chief structures and functions of the body and its viscera; the emergency treatment of contusions, wounds, hæmorrhages, fractures, dislocations, sprains, ruptures, burns, and accidents caused by electricity; what to do before the doctor arrives in cases of frost-bite, drowning, suffocation, syncope, heat stroke, and various kinds of poisoning; methods of transport; and the broad principles of lay nursing. We are a little surprised that the volume contains no index; an emergency book of this sort should be made such that reference to any point may be as easy and rapid as possible; it ought to be indexed in a very thorough and detailed fashion, and we regret that this has not been done. Nevertheless, the publication must be very helpful to all who enter for St. John's Ambulance Association classes, and, indeed, to every household. Its price places it within the reach of all.

First Aid to the Injured and Sick: An Advanced Ambulance Handbook. By F. J. Warwick, B.A., M.B.(Cantab.), M.R.C.S., L.S.A., and A. C. Tunstall, M.D., F.R.C.S.(Ed.). Fifth edition. Pp. 252. 234 figures. (Bristol: John Wright and Company, 1908. Prices 1s. net, paper; 2s. 6d. net, limp cloth.)

One of the best features of this book is the abundance of clear illustrations, which enable the ambulance student to see at once what should be done under the different conditions described in the letterpress. The chapter on transport of the sick and injured has been considerably extended in this edition, and fourteen new illustrations add greatly to the ease with which it can be understood. The diagrams make it clear at once how bodies can best be carried when no stretcher is at hand; how insensible persons may be conveyed down ladders in case of fire, and so forth. The illustrations of arterial compression for hæmorrhage from each of the various surface arteries of the body are very clear, too. The tables of poisons, with their immediate symptoms and the antidotal and other treatment required, are such that reference to them in emergency cases can be made with all rapidity. There are other books, such as that translated by Princess Christian, which should be read by the absolute beginner; but for lay persons who are seriously studying "First Aid" it would be difficult to find a better book than this one.

### HYGIENE.

Consumption: Home Treatment and Rules for Living. H. W. Crowe, M.D. Pp. 36, with chart. (Bristol: J. Wright and Co. 2nd Edition. 1s. net.)

This booklet is just the thing to recommend to a patient of some leisure who cannot be induced to go to a sanatorium. Too much has been written on this subject by those with inadequate experience of such places, but there is no sketchiness about Dr. Crowe's exposition, in plain language, of the principle of optimisation of exercise by means of the rectal temperature. Hot sunshine, however, is not good for consumptives, and breathing exercises, as Wright has shown, may produce auto-inoculation. But as a whole the book quite meets the case its title indicates.

School Hygiene. By R. A. Lyster, M.B., D.P.H. B.Sc. Lond. Pp. viii. + 360. (London: W. B. Clive. University Tutorial Press. 3s. 6d.)

The University Tutorial series has a reputation for capable contributors and carefully prepared work, which this manual well sustains. Its aim is to give teachers such information as will enable them to act as intelligent assistants to the school doctor. The range, therefore, is wide, covering not only hygiene, but also some physiology and household medicine, and naturally affording a loophole or two for criticism. The circumstances under which Galton grates may produce carbon monoxide might have been mentioned. The statement that breathing exercises purify the blood is tacitly endorsed, although Haldane condemns them as useless in this respect, because it is the proportion of carbon dioxide in the blood, on the contrary, which automatically regulates respiratory activity. In the chapters (excellently illustrated) on posture and school fittings no allusion is made to the student's instinctive lowering of the head in order to promote cerebral vascularity. Occasionally, too, the author expresses his dissent from authorities like Dr. Kerr in forcible terms. As a small but reliable compendium of a subject of much present and future interest, this book should have a wide circulation.

Third Annual Report of the Henry Phipps Institute for the Study, Treatment, and Prevention of Tuberculosis. (Philadelphia: Henry Phipps Institute. Pp. 410.)

Something very like the ideal plan for the collective study of urban tuberculosis seems to have been hit upon by Dr. Flick and his staff of colleagues at the Henry Phipps Institute of Philadelphia. A score of physicians, with laryngologists, pathologists, and other special workers, aided by nurses and lady visitors, share in the treatment of appropriate cases. Each, at monthly meetings, exchanges views and experiences with the rest, and each contributes to most carefully detailed records which are summarised annually. The findings in the present report are very convincing. It is stated *inter alia* that absence of bacilli in the sputum is of no value at all in diagnosis; that unfortunately it is very doubtful whether serum treatment benefits; that much rhinological defect is to be found amongst consumptives; and that tubercle bacilli may be excreted by the kidney. All these points (taken almost ordinally) agree well enough with conclusions already mooted or established elsewhere. Some novel results are that the Irish-American and Irish-German crosses especially show a phthisical predisposition, and that *B. pseudo-diphtheriæ* occurs frequently in hæmorrhagic cases, both in the lung and in the sputum. Although the various papers perhaps nowhere reach the highest standard of performance (a verdict sometimes warranted even in the case of a volume of German archives), it is clear that under none too favourable conditions much unselfish hard work is being done amongst the poor, and many most valuable data accumulated. A peculiarity of American note-taking is to write the date so that, for instance, 12/5/07 stands for December 5, not May 12, of last year.

### BACTERIOLOGY.

A Laboratory Handbook of Bacteriology. By Rudolf Abel. Translated from the tenth German edition by M. H. Gordon, M.A., M.D., with additions by Dr. A. C. Houston, Dr. T. J. Horder, and the Translator. Cap. 8vo. Pp. 224. Not illustrated. (Oxford Medical Publications; London: Henry Frowde and Hodder and Stoughton. Price 5s. net.)

This little pocket-book is "specially intended in the first place for the use of the physician and the veterinary surgeon," but "it will also help the apothecary and the chemist and those of other callings, as it includes what is necessary for their requirements." It seems to us, however, that it is essentially a handbook for the specialist in bacteriology rather than for anyone else. It is not a book from which bacteriology could be learned, but it is a handy compendium of bacteriological methods to which a laboratory worker could quickly refer for the verification of some point or detail in technique that for the moment had escaped the memory. Nutrient media are described at length, with their methods of production and the organisms for which each can be used. The best kinds of nutrient media, methods of culture, and staining processes are given for each of five and twenty different micro-organisms. There are descriptions of the ways to inoculate animals, and of how to examine these same animals post-mortem. Methods of preserving cultures and so forth are discussed, and there are special chapters upon the technique required for the bacteriological examination of water, milk, shellfish, vegetables, sewage, soil, dust, and air. There is a moderate index, and at the end of the book there are a number of blank pages for memoranda. We think there is a wonderful amount of information condensed into a small space, and bacteriologists will doubtless find the publication handy and of use; there are other books, however, which will be of far greater service to the general practitioner.

### STRAY NOTES.

The Romance of Medicine. By R. C. MacFie, M.A. Aberd., M.B., C.M. Pp. 312. (London: Cassell and Co. Price 6s.)

A competent popular account of the history of medical progress is the sort of book some patient will be sure to ask one's opinion of sooner or later. Not only for this reason, however, or because the author's preface recommends it to the profession, should one spend a couple of evenings in looking this little work over and realising afresh one's position as heir of the ages. The work of masters like Galen and Harvey is well described, but there is not much about John Hunter, and many will think too little has been made of the part Sir Patrick Manson played in connection with mosquitoes and malaria. Dr. MacFie has literary aptitude, and writes a good narrative; but the tone of some—not all—of his interpolations is curiously conversational and inappropriate. People in the mood to read about Hippocrates or Harvey, for instance, do not want regaling with quotations from "The Bab Ballads" or humorous references to current topics like the Suffragettes.

Alcohol. By H. E. Jones, M.B., C.M. Pp. 95. Seven illustrations. (Glasgow: Angus Macintyre. Price 3s. net, leather, or 2s. net, cloth.)

The much-vexed question of alcohol may be approached from many standpoints, and we have before us quite a novel way of dealing with the subject. The little book is not a scientific treatise, nor does it contain any statistics. It is a drawing-room address written in simple language, and it can be read in less than an hour It is not a rabid tirade against drink by an ardent teetotalist, for the good effects of the use of alcoholic beverages in moderation under certain conditions are pointed out, as well as the very bad effects of their use in other ways. It is clear that the author is, upon the whole, an opponent to the use of alcohol, but he does not give one the impression of being bigoted either way. The subject is dealt with in four parts, the most interesting of which is the first. This consists of word-pictures—vivid enough—illustrating the uses and abuses of alcohol, the good or bad effects it has had upon the different individuals depicted by the writer. The pictures could only have been drawn by a medical man. The other three parts are devoted to a popular account of alcohol itself and of the way it produces the different effects on the individuals described in the first part. It is a clever conception, carried out with some success; it is quite distinctly an address for the laity, however, or for the physician in his lay capacity. The key to the idea is given in the author's concluding paragraph, most of which we can thoroughly endorse: "Teach the young to love and obey their parents; teach them the physical, mental, and moral effects of Alcohol; teach them to say *no* and to *mean* it; teach them to . . . respect themselves, and you will in time close the public-houses, not by reducing licenses only, but by bringing up a race who will pass public-houses by as though they did not exist. Begin early, lead them firmly but kindly, guide them, not by the teacher and the rod, but at the fireside with mother's love. Home influence will do more good than many temperance lectures."

The New Ethics. By J. Howard Moore. Pp. viii.+216. (London: Ernest Bell. 1907.)

Wendell Holmes once sighed for a critic who should give him an appreciation of a book in an epithet and a wink. The proper verdict here would be a smile, amused but kindly. The new ethic is extreme humanitarianism: the author would scarcely refuse a blood-sucking fly "the little drink of sweet red wine" it craves. After this we know what to expect on vivisection. It is all very naïve, and rather attractive, with its modest little bibliography, its generous denunciation of fur-trapping barbarities, and its unconsciously slipshod use of transatlantic colloquialism. In his next invective Mr. Moore should try to exercise more discrimination.

